# How Systemic Barriers Can Impact Health Inequities When Facing Climate Change Stressors: A Scoping Review of Global Differences

**DOI:** 10.1029/2024GH001272

**Published:** 2025-06-18

**Authors:** Ainslee Wong, Tuyet‐Mai H. Hoang, Victoria Ferrara, Thanh H. Nguyen

**Affiliations:** ^1^ Department of Health and Kinesiology University of Illinois Urbana‐Champaign Urbana IL USA; ^2^ School of Social Work University of Illinois Urbana‐Champaign Urbana IL USA; ^3^ Department of Civil Engineering University of Illinois at Urbana‐Champaign Urbana IL USA

## Abstract

The objective of this scoping review is to explore the systemic barriers that impact health inequities among vulnerable populations (e.g., racial/ethnic and gender groups, people with disabilities, refugees, immigrants, elders, young children, agricultural and fishery workers, and low‐income individuals) when facing climate change stressors. We conducted an extensive review using nine search engines, which yielded 21 publications that focused on the health outcomes and barriers on the topic of climate change among vulnerable populations. Our findings indicated that poverty is the largest challenge preventing people from adequate health access and achieving positive outcomes, particularly for vulnerable populations globally. In addition, institutional and systemic barriers also differ based on regional differences, which suggests that health inequities are context dependent. Our scoping review has implications for (a) enhancing the effectiveness of climate change mitigation strategies and (b) addressing the healthcare barriers of vulnerable populations based on country‐specific challenges.

## Key Points

- A successful and sustainable climate mitigation effort needs to address health inequities when facing climate stressors
- The reduction of healthcare inequities is dependent on examining country‐specific healthcare barriers that impact vulnerable populations
- A recommendation is made to explore global healthcare barriers of vulnerable populations to ensure effective climate mitigation strategies

## 1 Introduction

Since the 20th century, the rise in Earth's average surface temperature has contributed to regional and seasonal temperature extremes, such as reductions in snow cover and sea ice, intensified heavy rainfall, and changes in plant and animal habitat boundaries (Lindsey & Dahlman, 2024). With current warming trends increasing three times faster than in earlier decades, it is evident that the pace of global warming continues to accelerate (Lindsey & Dahlman, 2024). More climate change–related stressors such as severe drought, heat, flooding, hurricanes, and water salinity impact daily life globally and have resulted in major health‐related consequences, such as food insecurity, spread of disease, and lack of access to clean water and air (National Oceanic and Atmospheric Administration, 2021). The goal of climate change mitigation efforts as outlined by the United Nations is to avoid the worst climate impacts on our living environment and maintain a livable climate for all; this emphasizes the lasting impact of climate stressors on human health outcomes. As we already have learned from previous natural disasters like the recent Hurricane Milton in the United States or Typhoon Yagi in Vietnam, health inequities are at the forefront of adverse human health outcomes as extreme weather events become more common (National Academies of Sciences, Engineering, and Medicine, 2017). Furthermore, health is a key focus of three major international conventions: the 2030 Agenda for Sustainable Development, the Paris Agreement under the United Nations Framework Convention on Climate Change, and the Sendai Framework for Disaster Risk Reduction. These conventions emphasize the risks and opportunities associated with building climate‐resilient communities, underscoring the importance of equity and sustainability in enhancing community environmental resilience. Consistent with this focus on living environment, equity, and impact on health outcomes, our review aims to emphasize that addressing health inequities should be a core tenet in all climate change adaptation efforts.

Climate impact on infrastructure and the environment result in negative health outcomes through direct (e.g., lack of clean water and air, food/housing/employment insecurity, injuries) or indirect (e.g., contamination increases, lasting respiratory and cardiac conditions, and prevalence of food/water borne illnesses) contact (National Institute of Environmental Health Sciences, 2022). While the impact of climate change affects everyone, small island nations, the Global South, and other developing countries are the most vulnerable (Yohe et al., 2006; Yore et al., 2023). Given that countries can have major differences in the types of climate‐induced risks and sociocultural and economic variations, the impact of climate change stressors can be country‐specific regarding the types of health outcomes and inequities vulnerable populations may face (Chambers, 2020).

To effectively address health inequities and adverse outcomes caused by climate change, countries must tailor their mitigation strategies to the specific risks they encounter and consider how these risks impact different populations. This approach is crucial because health inequities, defined as systemic health variations in the health of different populations, often arise from social factors and barriers at community, institution, and system levels (Johannes et al., 2019; World Health Organization, 2018). The mechanisms by which these barriers operate are specific to the historical, political, and geographical context of a region, and to date, there is a gap in literature examining the manifestation of context‐specific healthcare barriers that drive health inequities among vulnerable populations (Edwards & Di Ruggiero, 2011). The goal of climate change action is to avoid the worst of climate change impacts and maintain a livable climate for everyone, and it is imperative to include a plan for addressing healthcare barriers and reducing inequities in climate change mitigation strategies. While previous studies have identified systemic barriers to healthcare, our review uniquely addresses both these barriers and the access issues faced by vulnerable populations, which are essential to exploring the mechanisms of these barriers in detail and with geographic context differences. In our study we aim to conduct this step by examining the results of previous studies that explore the effects of climate change exposure on the mental and physical health outcomes of vulnerable populations. Second to this aim, we explore the systemic barriers and drivers to healthcare inequities that these studies conclude result from climate change stressors. Due to the review nature of this study, our analysis is limited to the conclusions and inherent strengths and weaknesses of previous studies. This study also provides recommendations to consider in drafting and implementing climate change mitigation approaches.

### 1.1 Current Study

The objective of our study is to utilize the inferential findings of previous studies to identify healthcare barriers and explore their impact on health inequities among vulnerable populations when facing climate change stressors. Our study reviews existing studies and provides a checklist of considerations for addressing the health needs of vulnerable populations during a time of climate crisis. The findings provide insight on (a) how systemic factors play a role in the health access and inequities among vulnerable populations, (b) differences in healthcare barriers of vulnerable populations varied by geographic and sociocultural context, and (c) potential considerations to address healthcare barriers and reduce inequities for vulnerable population in climate change mitigation plans. We identified two research questions:

1. How does exposure to climate change stressors contribute to physical and mental health inequities among vulnerable groups, and how does this differ by region?
2. For vulnerable populations impacted by climate change risks, what are the social, institutional, and systemic barriers that contribute to health inequities and lack of access?

## 2 Materials and Methods

This scoping review utilized the framework and methodology developed by Arksey and O’Malley (2005) and recommendations from Munn et al. (2018). Our process involved (a) determining the research questions; (b) identifying relevant studies; (c) selecting studies based on review criteria; (d) evaluating the data; and (e) compiling, summarizing, and reporting the results.

### 2.1 Identifying Studies

To identify relevant literature, we utilized nine search engines: Google Scholar, PSYCinfo, EBSCO, PubMed, Agricola, CABI Global Health, CINAHL, Web of Science, and MEDLINE. Keywords included health disparities, climate change, mental health, physical health, access, inequalities, and forms of discrimination. A full list of search terms can be found in Table 1. We excluded duplicate studies, dissertations, theses, and/or studies that did not address a combination of environmental, health, and accessibility outcomes. Included studies encompassed original research published in English that specifically investigated the impact of climate change on health outcomes and healthcare accessibility.

**Table 1:** Climate Change Keywords

Health disparities	Climate change	Access	Inequalities	Form of discrimination
“Health” OR “disease” OR “illness” OR “well‐being” OR “robustness” OR “liveliness” OR “weakness” OR “debilit*” OR “exposome” OR “determinant of health” OR “exposure” OR “morbidit*” OR “mortalit*” OR “life expectancy” OR “death” OR “mental” OR “mental stability*” OR “mind” OR “healthy” OR “psychological” OR “balance” OR “emotional” OR “physical” OR “state of health” OR “condition”	“Climate change” OR “climate crisis” OR “interchange” OR “global warming” OR “Global Heating” OR “Climate justice” OR “climate emergenc*” OR “climate exposure” OR “Climate Breakdown”	“Access” OR “clinical intake” OR “intake” OR “admittance” OR “diagnosis” OR “get help” OR “drivers of health and equit*” OR “admission” OR “entrance” OR “gateway” OR “means of access” OR “Barrier”	“Inequality*” OR “disparit*” OR “inequit*” OR “unequal” OR “discrim*” OR “prejudic*”	“Race” OR “Racism” OR “ethnic*” OR “skin color” OR “skin colour” OR “skin tone” OR “BME” OR “BAME” OR “ethnic minorit*” OR “environmental racism” OR “Migrant” OR “refugee*” OR “asylum seek*” OR “immigrant*” OR “immigrat*” “discrim*” OR “prejudic*” OR “xenophobi*” OR “antisemt*” OR “anti semt*” OR “islamophobia*” OR “caste” OR “indigen*” OR “aborginial” OR “first nation*” OR “first people*”

### 2.2 Literature Section

Initially, our search produced 26,249 records. To minimize bias within our literature selection, we followed recommendations from McDonagh et al. (2013), which included: (a) developing clear and specific inclusion and exclusion criteria to reduce ambiguity during study selection, (b) assigning multiple reviewers to independently assess each study's eligibility, and (c) implementing a two‐stage screening process—initially excluding studies based on title relevance, followed by a more detailed abstract review—to eliminate clearly ineligible articles early and allocate more thorough evaluation to studies with greater inclusion potential. In cases of disagreement over eligibility, the authors met to discuss discrepancies and reach a final decision. Three authors (AW, TF and TMH) read all of the titles manually and individually. All three authors met to discuss disrepancies and make final decision. During review of article titles, we excluded duplicate articles (*N* = 280), articles published in a language other than English (*N* = 8), and articles whose titles were of insufficient relevance (e.g., those where it was clear the target species was not humans or where there was a lack of connection to one or more of the five key categories of keywords) (*N* = 25,589). After these exclusions, all authors read every abstract of the remaining 372 articles to determine study eligibility. In cases of disagreement over eligibility, the authors met to discuss discrepancies and reach a final decision.

**Figure 1:**
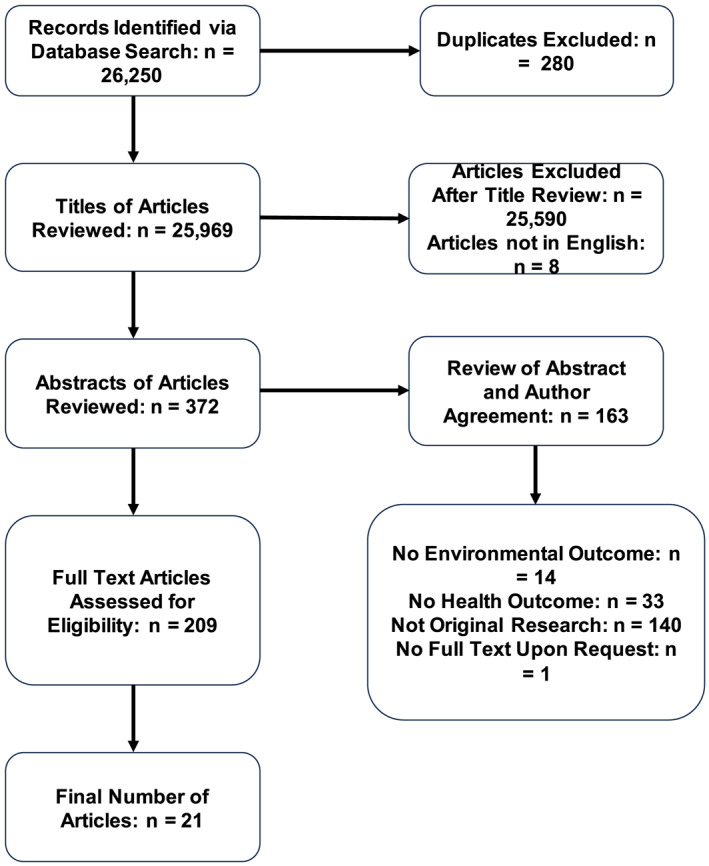
Literature selection process.

Studies that did not address environmental outcomes and health outcomes were excluded. Inclusion criteria for the study were (a) evaluated a health outcome impacted by a climate change stressor, (b) addressed health accessibility issues due to systemic and structural barriers, (c) written in English, (d) target population was a vulnerable group, and (e) inclusion of original research and not a review or opinion. For the purpose of this study, vulnerable groups were defined as populations that were deemed vulnerable due to a particular social identity or occupation (e.g., racial/ethnic minority, low‐income groups, marginalized gender identities, refugees, immigrants, and/or agricultural workers). Systemic barriers were defined as obstacles for certain groups that are embedded within the overarching structure and cultural norms of a society, and structural barriers were defined as obstacles created for certain groups of people through the policies, procedures, and structures of an institutional entity (Bonilla‐Silva, 1997). After determining the eligibility of 21 research articles, we utilized a standardized checklist of 10 questions to evaluate the various research methods used in each study. This checklist consisted of four questions to assess external validity (e.g., was some form of random selection used to select the sample OR was a census undertaken?) and six questions to assess internal validity (e.g., were data collected directly from the subjects (as opposed to a proxy)?). All articles at this stage were found to have sufficient scientific rigor for inclusion and we provide this list of assessment questions in the supplemental material. Our final sample included 21 original research articles (Figure 1). Of these, 18 studies provided sample sizes, for a total of 10,262,140 participants. The remaining three studies did not report sample sizes.

### 2.3 Charting and Summarizing Data

To effectively evaluate each study based on our research questions, we created a standardized worksheet. This worksheet consisted of one research question in each column, where relevant details extracted from each study were charted by all authors. Each author evaluated each study independently, and 18 meetings were held to discuss variations and reach a consensus. The first research question focused on investigating differential mental and/or physical health outcomes that resulted from climate change–related stressors including natural disasters, extreme weathers, and pollution in air and water. The second question focused on examining social, systemic, and/or structural barriers that impacted a population's access to healthcare. We also created a standardized table to summarize study characteristics (see Table 2).

**Table 2:** Study Characteristics

Author(s), year	Methods	Country	Vulnerable population studied	Climate change stressor	Climate risk	N	Aim/Objective of study	Outcomes
Studies Conducted in North America								
Adepoju et al. (2022)	Mixed Methods	USA	African Americans	Climate‐ Related Natural Disasters	Increased Disease Burden	N/A	To investigate the disproportionate health impacts of climate change on people of color.	African American communities experienced a greater disease burden, greater vulnerability, and higher community‐level risk factors during disaster events. Social connectedness enhanced resiliency during disasters. Prior experience with nature disasters also increased the likelihood that communities were prepared.
Albahsahli et al. (2023)	Qualitative	USA	Refugees	Climate‐ Related Natural Disasters	Hypertension	67	To find links between structural drivers of climate change and health inequities for refugee populations.	Participants experienced harsher weather conditions, particularly an increased exposure to extreme heat and cold. A lack of financial resources impacted their ability to access safe housing and resources such as heating in temporary resettlement sites.
Behrman and Weitzman (2016)	Quantitative	Haiti	Women	Earthquakes	Adverse Consequences to Women's Reproductive Health	43,918	To explore the impact of the 2010 Haiti earthquake on women's reproductive health.	Exposure to severe earthquake intensity reduced women's access to condoms and left them with unmet family planning needs. Additionally, women's ability to negotiate the use of a condom with partners decreased.
Flores et al. (2020)	Quantitative	USA	Elderly	Hurricanes	Increased Physical and Mental Health Problems	403	To assess health disparities in physical health, mental health, and healthcare access after Hurricane Harvey.	Physical health issues disproportionately impacted people who did not evacuate during the hurricane. Posttraumatic stress was more likely in non‐Hispanic Black people, elderly people, and people who lost jobs during the hurricane. Access to healthcare was also limited for people with disabilities and people who lost jobs.
Guirguis et al. (2018)	Quantitative	USA	Low‐Income Groups	Climate and Temperature Variability	Increased Disease and Illness	853,232	To analyze hospitalization and temperature data in San Diego County in order to document health outcomes on a city‐wide scale.	Coastal regions had a greater prevalence of multiple illnesses at lower temperatures when compared to their inland counterparts. In coastal areas where air conditioning is not prevalent, there was an increase in heat‐related morbidity.
Kim et al. (2021)	Quantitative	USA	Pregnant Women	Extreme Temperatures	Maternal Hospitalizations During Pregnancy	2,240,000	To explore the link between exposure to extreme temperatures and the risk of maternal hospitalization during pregnancy.	While adverse outcomes rose for all groups during extreme temperatures, Black women were more severely impacted compared to White women.
Mendez et al. (2020)	Qualitative	USA	Refugees and Immigrants	Fire	Unsafe Working Conditions	30	To assess the gaps in emergency response to the Thomas fire, a disaster in California's Ventura and Santa Barbara counties.	The fire created barriers to transportation and safe housing. Employment was also impacted, as many people could not work or continued to work in dangerous conditions (in the case of farmworkers). Communication issues were apparent at the beginning of the crisis, since warnings and guidelines were primarily published in English only.
O’Neill et al. (2005)	Quantitative	USA	African Americans	Heat	Mortality	684,847	To examine if air conditioning (AC) prevalence had an effect on heat‐related mortality racial disparities.	Heat‐associated mortality was higher in Black populations, and Black households had a 5.3% higher heat‐related mortality when compared to White households. Central AC prevalence in White households was more than double that in African American households. For each 10% increase in AC prevalence, heat‐associated mortality decreased by 1.4%.
Schmeltz et al. (2016)	Quantitative	USA	Women	Heat	Heat‐Related Illnesses (HRIs)	73,180	To explore hospitalizations for HRIs and their associated costs.	Participants with HRIs were more likely to be male and/or reside in the lowest zip code income quartile. Costs for HRI hospitalizations were greater for Black, Hispanic, and Asian Pacific Islander groups when compared to White groups. People with private insurance had lower costs than those who used Medicaid and/or Medicare.
Studies Conducted in Oceania								
Green et al. (2015)	Quantitative	Australia	Indigenous Groups	Temperature Extremes (Cold)	Respiratory Disease	N/A	To explore the difference in respiratory disease hospital admission rates for Indigenous and non‐Indigenous Australians during periods of temperature extremes.	During cold days, there was an increase in non‐Indigenous children's admissions for acute respiratory disease but not Indigenous children's. There was not a consistent pattern in admissions that occurred on hot days.
Loughnan et al. (2010)	Quantitative	Australia	Low‐Income Groups	Temperature Extremes (Heat)	Acute Myocardial Infarction (AMI) Admissions	871	To analyze the effect of weather on people with pre‐existing cardiac disease through the lens of a sociodemographic perspective.	The most disadvantaged and least disadvantaged areas had threshold temperatures above which AMI increased. With temperatures above a threshold of 30°C, there was a 10.8% increase in AMI admissions, and patients admitted were predominantly younger than 70 years old. With temperatures above a threshold of 27°C for 3 days, there was a 37.7% increase in admissions and more people between the ages of 55 and 74 were impacted.
Murphy et al. (2023)	Qualitative	Fiji	Youth	Climate‐ Related Natural Disasters	Reproductive Health	21	To explore the experiences of Fijian youth in fulfilling sexual and reproductive health needs during natural hazards and disasters.	Fijian youth had inconsistent access to sexual/reproductive health knowledge, which encouraged the use of unreliable sources. Elders tended to gatekeep sexual/reproductive health knowledge, and the sexual/reproductive health needs of Fijian youth were not a priority for them. Limited access to resources like food, water, and private bathrooms increased the use of transactional sex to fulfill needs.
Studies Conducted in Europe								
Salvador et al. (2023)	Quantitative	Spain	Elderly	Heat	First Cardiovascular Event (CVE) Incidence	6,514	To explore associations between heat and the incidence of a first acute CVE.	Non‐Spanish residents and males were more affected by the heat. Older adults had a slightly higher risk for a first acute CVE in the summer. After an extreme heat day, risk of suffering a first CVE increased by 15.3%
Studies Conducted in Asia								
Hu et al. (2019)	Quantitative	China	Rural Groups	Extreme Temperatures (Heat and Cold)	Mortality	6,000,000	To investigate if temperature–mortality relationships differ for populations in urban and rural areas of China.	Low and high temperatures in urban and rural areas were both associated with an increased mortality risk, but rural areas had a higher related risk. During cold periods, rural areas had a higher all‐cause mortality rate than urban areas.
Kabir et al. (2016)	Qualitative	Bangladesh	Agricultural Workers	Cyclones	Increased Disease Prevalence	N/A	To explore the experiences of people who lived on the coastal areas of Bangladesh during cyclones Sidr and Aila.	Climate change negatively impacted the health and socioeconomic status of participants. Particularly, livelihood patterns were disrupted, which contributed to an increase in unfavorable health conditions.
Lin et al. (2022)	Quantitative	Taiwan	Agricultural and Fishery Workers	General Climate Change	Increased Disease Prevalence	352,520	To longitudinally investigate the health status of fishery workers in comparison to counterparts with similar socioeconomic status and occupation.	Compared to farmers, fishery workers were more likely to be diagnosed with a myriad of illnesses (e.g., hypertension, diabetes, peptic ulcers, etc.). Compared to farmers and employed workers, fishery workers were more likely to suffer from cardiometabolic diseases, mental illness, infection, malignancy, etc. Fishery workers had an average monthly income less than 800 USD, while half of employed workers had an average monthly income greater than 800 USD.
Thamarapani (2021)	Quantitative	Indonesia	Youth	Climate‐ Related Natural Disasters	Developmental Health	1,262	To examine the effects of exposure to natural disasters on child health.	Children in households that experienced economic loss were 13% more likely to have stunted growth. Exposed girls were 7% more likely to be stunted after experiencing a natural disaster. There was no effect on boys.
Studies Conducted in Africa								
Ajibade et al. (2013)	Mixed Methods	Nigeria	Women and Low‐Income Groups	Floods	Mortality, Illness, and Injury	453	To investigate the impact of flooding on women's health and lives in Lagos.	Women did not see their experience of flooding events as gendered. Women in low‐income neighborhoods experienced a greater impact from flooding and a slower recovery time than other social groups.
Berrang‐Ford et al. (2012)	Qualitative	Uganda	Rural Groups and Ethnic/Racial Minorities	General Climate Change	Increased Prevalence of Disease	174	To assess health vulnerabilities utilizing a bottom‐ up approach in rural Uganda.	The greatest challenge faced by participants was a consistently poor health status and greater burden of disease due to climate change. Livelihood dislocation adversely impacted the health of Batwa participants because they no longer had access to traditional forest products.
MacVicar et al. (2017)	Qualitative	Uganda	Rural Groups	Variability in Seasonality and Weather	Adverse Pregnancy Outcomes	26	To compare the perinatal experiences of Indigenous (Bakiga) and non‐Indigenous (Batwa) mothers in relation to seasonality and weather.	Batwa and Bakiga participants reported similar experiences of seasonality and weather, with dry seasons being associated with the greatest food scarcity and lower quality of food, both of which were perceived to be associated with negative infant outcomes. Barriers to accessing healthcare, such as transportation, were also discussed. Between the two groups, key informants believed that the Batwa population faces greater severity in experiences than the Batiga population.
Studies Conducted in South America								
Da Mata et al. (2023)	Quantitative	Brazil	Pregnant Women	Dry Seasons	Birth Outcomes	4,622	To estimate the effects of prenatal exposure to cisterns on birth outcomes.	Birth weight increased when pregnant women had access to cisterns during early pregnancy, particularly for mothers who were more educated.

Table 3 shows a checklist guide for researchers, policymakers, and other relevant stakeholders to utilize in addressing the effects of climate change on vulnerable populations with respect to different countries' needs. Our guideline checklist highlights country‐specific climate change stressors and systemic barriers that exacerbate climate‐related health inequities. Furthermore, the reference articles and questions are provided as a guide to addressing the impact of climate change stressors in each country's unique sociopolitical context.

**Table 3:** Recommendations for Addressing Institutional and Structural Issues When Creating Climate Change Mitigation Efforts

Continent	Institutional/Structural issues to consider in climate change mitigation efforts	Recommended standards of mitigation approach	Resources
North America	Poverty preventing access to healthcare resources	Address the need for food security and water management in communities who are at risk of deforestation and natural resource degradation.	(Climate Focus, 2023)
		Reduce emissions of methane, black carbon, and other short‐lived climate pollutants.	(Sun et al., 2022)
		Improve access to cleaner energy sources.	(Farghali et al., 2023)
Oceania	Poverty preventing access to healthcare resources	Reduce emissions of methane, black carbon, and other short‐lived climate pollutants.	(Münzel et al., 2020)
		Enhance food and water security.	(Misra, 2014)
		Create greater livelihood resiliency and socioeconomic opportunities for vulnerable populations.	(Mcleod et al., 2019)
Europe	Communication methods and strategies that account for cultural diversity and language differences within a population	Use accessible language.	(Rakhimov et al., 2022)
		Consider knowledge of the target population.	(Yore et al., 2023)
		Ensure comprehensive dissemination.	
Asia	Poverty preventing access to healthcare resources like air conditioning, water resources, and hospital care	Develop stronger social protection systems.	(United Nations Environment Programme, 2024)
		Create food systems approaches that are designed to sustainably support the livelihoods of vulnerable populations and enhance their food security.	(UNICEF, 2023)
		Strengthen adaptive capacity to impacts for climate change.	
Africa	Poverty preventing access to healthcare resources such as transportation and improving the safety of living environments.	Address the need for food security.	(Kemoe et al., 2022)
		Create economic and enterprise opportunities for youth.	(Lifering & Lacey, 2022)
South America	The lack of infrastructure to obtain clean water and lack of knowledge about the effects of clean water practices on the health of vulnerable populations	Develop hydraulic infrastructure.	(Rodrigues et al., 2023)
		Address water productivity and efficient irrigation practices.	(Da Mata et al., 2023)
		Obtain financing to operate and maintain water sanitation services, particularly for rural populations.	

## 3 Results

Our study included 21 published articles on the topic of climate change stressors, health inequities, and accessibility issues. The majority of studies (*n* = 9) were conducted in North America, with the remaining studies conducted in Asia (*n* = 4), Oceania (*n* = 3), Africa (*n* = 3), Europe (*n* = 1), and South America (*n* = 1). Out of the original articles included in our study, 13 used quantitative methods, six used qualitative methods, and two used mixed methods. The most common data source for quantitative studies was census data (*n* = 5), and the most common research design was a retrospective cohort study (*n* = 6). For the qualitative studies, the most common research design was semi‐structured interviews (*n* = 3). Both mixed methods studies utilized a combination of focus groups and surveys. All studies across all categories were observational. The methodological diversity of these studies highlights the range of strategies employed to examine the complex relationship between climate change stressors and health outcomes; and understanding the strengths and limitations of these methods is essential for interpreting their findings accurately. For instance, large epidemiological studies that draw from census data or medical record databases often benefit from more representative samples, reducing selection bias; however, they may lack detailed information on data quality and potential confounding variables (Thygesen & Ersbøll, 2014). Likewise, qualitative methods such as semi‐structured focus groups and interviews allow for comparative analysis through a core set of questions while offering flexibility in discussion; however, they are limited by potential selection bias and the influence of participants' unconscious or recall biases (Diefenbach, 2009). The observational aspect of all reviewed studies within this study also presents a limitation as causation cannot be inferred. Therefore, the contextual strengths and methodological constraints of each of these studies should be considered when interpreting this review. Furthermore, most studies included populations deemed vulnerable based on demographic factors. Additional study characteristics can be found in Table 2. The results are presented in two categories: countries in the Global North (*n* = 11) and countries in the Global South (*n* = 10), to highlight the impact of systemic factors across different regions. For this review, countries in the Global North were defined as developed and industrialized countries (i.e., the United States, Spain, and Australia; Kowalski, 2021). Countries in the Global South were defined as developing countries (i.e., China, Taiwan, Haiti, Fiji, Brazil, Uganda, Nigeria, Indonesia, and Bangladesh; Kowalski, 2021). Additionally, results are broken down by continent to explore the sociocultural context.

### 3.1 Research Question 1: Exacerbation of Health Inequities From Climate Change Stressors

**Figure 2:**
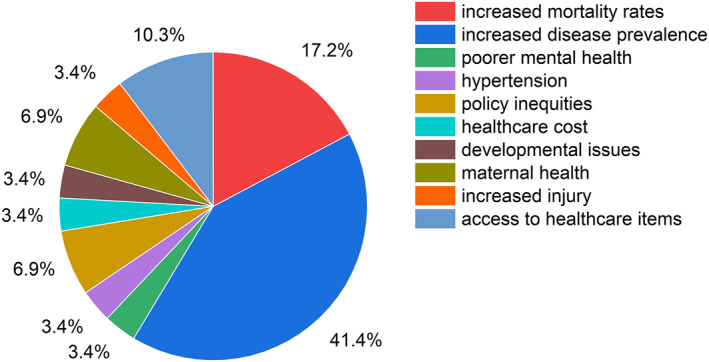
Health inequities impacted by climate change stressors.

We examined studies to discover whether certain health inequities are exacerbated by specific climate change stressors. Overall, the increased prevalence of disease emerged as the most frequently discussed health inequity, drawing attention to the urgent need for action in addressing this pressing issue. Following closely behind were rising mortality rates, which further underscores the severity of the challenges posed by health disparities. These discussions emphasize not only the immediate impact on vulnerable populations but also the long‐term implications for public health systems. For more detailed insights into how climate change exacerbates health inequities on a global scale, please refer to Figure 2. This figure provides a global overview of health inequities impacted by climate change, illustrating the interconnectedness of environmental changes and health outcomes. Understanding these dynamics is crucial for developing effective strategies to mitigate the adverse effects of climate change on health equity worldwide. Furthermore, the following sections are organized by continent to highlight the similarities and differences in climate change stressors experienced across various regions.

#### 3.1.1 Global North

The majority of studies (*n* = 9) conducted in the Global North found that vulnerable populations were likely to experience an increased prevalence of diseases and illnesses (e.g., respiratory disease, malaria, cardiometabolic disease, hypertension, heat‐related illness, diabetes, and mental illness) and severity of health outcomes (e.g., increased acute myocardial infarctions, mortality, hospitalization, and adverse pregnancy outcomes) when exposed to climate change stressors, compared to periods without such stressors. High heat was a particular concern for countries in the Global North, with 36% (*n* = 4) of studies citing heat as the primary climate change stressor that were associated with adverse health outcomes (e.g., increased mortality rates, increased prevalence of heat‐related illnesses, increased incidence of a first cardiovascular event, and increased myocardial infarction admissions).

##### 3.1.1.1 North America

Seventy‐five percent (*n* = 6) of North American studies conducted in the Global North found increased prevalence of disease and severity of adverse health outcomes. Particularly, a greater burden of disease and increased mental and physical health issues were associated with disaster events. Furthermore, one study found that populations without access to air conditioning were more vulnerable to exposure to increased temperatures and their associated adverse health outcomes. One example is Guirguis et al. (2018), which found that hospitalizations, heat illness, dehydration, and acute renal failure were more associated with populations who lived in the coastal regions of California where air conditioning was inaccessible during hot days. Thirty‐eight percent (*n* = 3) of studies based in North America saw an increased mortality rate related to climate change stressors, particularly in association with temperature fluctuations (Guirguis et al., 2018; O’Neill et al., 2005). For example, O’Neill et al. (2005) found that the presence of central air conditioning in the United States was more than double in White households than Black households, which could provide insight into the higher rate of heat‐related mortality in Black populations compared to White populations. Climate change stressors also increased the likelihood of poorer mental health (Flores et al., 2020) and inaccessibility to healthcare resources (Flores et al., 2020; Schmeltz et al., 2016). Loss of work (Flores et al., 2020) and policy inequities (Mendez et al., 2020) were also associated with climate change stressors related to natural disasters.

##### 3.1.1.2 Oceania

All Oceanic studies conducted in the Global North found a greater association between higher prevalence of disease and severity of adverse health outcomes (e.g., increased respiratory disease and acute myocardial infarction) among vulnerable populations in relation to an increased heat experience when compared to the general population. For example, Loughnan et al. (2010) found that the prevalence of acute myocardial infarction in the most socioeconomically disadvantaged areas and least socioeconomically disadvantaged areas in Australia tended to increase during periods of hot weather.

##### 3.1.1.3 Europe

One study found that an increase in heat was associated with greater adverse health outcomes and the prevalence of disease. Particularly, cardiovascular diseases and/or events that occurred in the summer were the most relevant. One example of this is Salvador et al. (2023), which found that the risk of experiencing an acute cardiovascular event increased for populations such as older adults and non‐Spanish residents after exposure to an extreme heat day in Spain.

#### 3.1.2 Global South

Eighty percent (*n* = 8) of studies conducted in the Global South found that climate change stressors were positively correlated with an increase in adverse health outcomes (e.g., stunted growth, adverse pregnancy outcomes, mortality, and workplace injury) and increased prevalence of disease (e.g., malaria, typhoid, cholera, kidney disease, hypertension, cardiometabolic disease, and skin disease). Structural damage (e.g., damage to homes, properties, and shops) and loss of work were also found to be associated with climate change disasters, with 20% (*n* = 2) of studies finding that vulnerable populations experienced one or both effects.

##### 3.1.2.1 Asia

Three of the four studies conducted in Asia found that the prevalence of disease and severity of adverse health outcomes were positively correlated with climate change stressors. Climate change effects related to these outcomes varied, including exposure to general climate change, increased temperature, and climate‐related disasters. For example, Lin et al. (2022) found that fishery workers in Taiwan were more likely to be diagnosed with a myriad of illnesses (e.g., hypertension, peptic ulcers, diabetes) when compared to farmers and employed workers because of the different level of exposure to climate risks. Natural disasters caused by climate change also increased the likelihood of infrastructure damage (e.g., shops, dams, and homes; Kabir et al., 2016), loss of work (Kabir et al., 2016), and developmental issues (Thamarapani, 2021) for vulnerable populations. Furthermore, 25% (*n* = 1) of studies found an increase in mortality rates due to rising temperatures.

##### 3.1.2.2 Africa

Sixty‐six percent (*n* = 2) of studies conducted in Africa saw a positive correlation between adverse perinatal health outcomes and climate change stressors. There are various documented climate stressors in Uganda such as the escalation of rising temperatures (Berrang‐Ford et al., 2012) and a general change in weather conditions (MacVicar et al., 2017). For example, MacVicar et al. (2017) found that mothers from a non‐Indigenous population in Uganda were more likely to experience negative infant outcomes due to increasingly drier seasons, which impacted their ability to secure nutritious food. One study also found that women in Africa were more likely to experience structural damage, loss of work, and increased injury after natural disasters (Ajibade et al., 2013).

##### 3.1.2.3 South America

One study found that an increase in general climate change stressors was associated with adverse health outcomes through access barriers to clean water. One example is Da Mata et al. (2023), who found that maternal and infant health in Brazil was significantly improved by access to clean water, which was correlated with increased birth weight.

##### 3.1.2.4 North America

One study conducted in the Global South found an increase in unmet family planning needs during natural disasters. An example of this is Behrman and Weitzman (2016), which found that earthquakes in Haiti reduced women's access to condoms and injectables and their ability to negotiate condom use with sexual partners, which contributed to an increase in unwanted pregnancies.

##### 3.1.2.5 Oceania

The one Oceanic study conducted in the Global South found that climate‐related natural disasters reduced vulnerable populations' ability to meet sexual health needs. For example, Murphy et al. (2023) found that legal equity and access to healthcare resources for Fijian youth was inhibited after experiencing a climate change‐related natural disaster.

#### 3.1.3 Discussion: Increased Prevalence of Disease, Illness, and Temperature Changes

Our findings support the focus on context‐driven factors when developing and implementing climate mitigation approaches for each country. To prevent or reduce the negative impact of climate stressors, we need to understand the specific climate change challenges of the targeted continent or country. For example, cardiovascular illnesses are more likely to increase in continents that experience an influx of extreme heat, while respiratory diseases are more likely to increase in continents that experience an influx of both extreme cold and extreme heat (D’Amato et al., 2014; Desai et al., 2023). Furthermore, the impact of fluctuating temperatures due to climate change has the potential to change weather patterns, which impacts the occupational health of agriculture workers and access to resources such as nutritious food and clean water (Gornall et al., 2010). When creating climate mitigation strategies, it is critical to situate the policy within the geographical climate of the region to effectively address climate change stressors and their impact on the health of vulnerable populations.

### 3.2 Research Question 2: Barriers That Contribute to Health Inequities

Countries' unique systemic, social, and/or structural barriers that impact access to healthcare were also assessed to determine how they contribute to climate‐related health inequities. For example, a lack of adequate transportation would be considered a systemic barrier, while the loss of public transportation due to a car‐centric culture would be considered a structural barrier. While we were unable to examine structural factors due to a lack of data, identifying specific systemic barriers within an area is crucial in determining their impact on health inequities because these barriers differ depending on the current geopolitical context of a country.

#### 3.2.1 Global North

**Figure 3:**
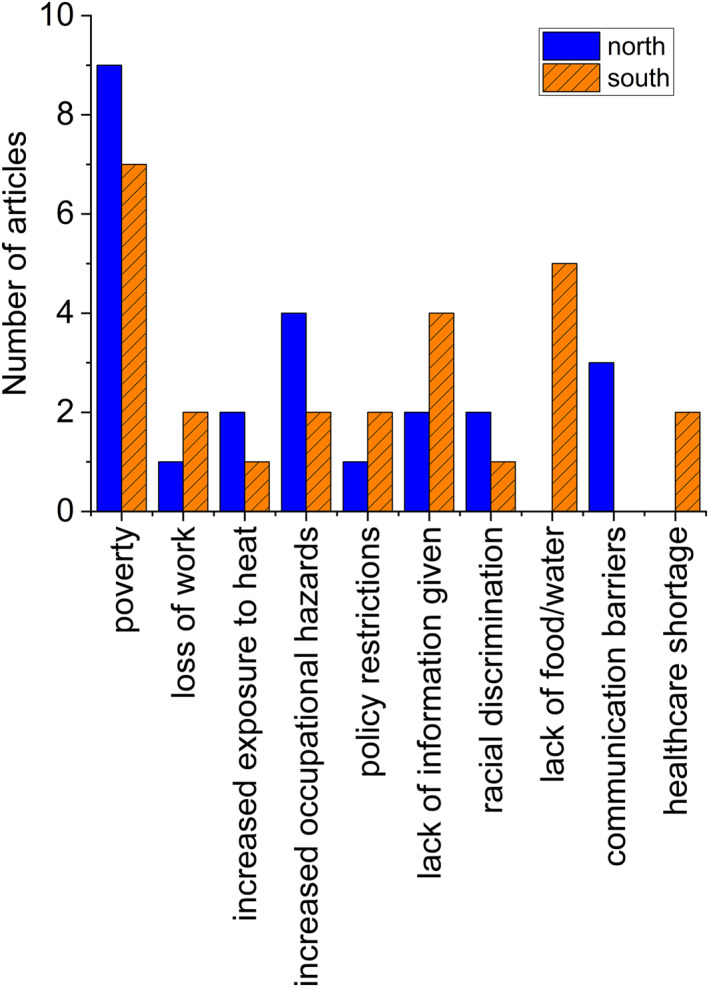
Barriers to healthcare access in the global North and global South.

Overall, nine of the 11 studies conducted in the Global North found that low income was a barrier to vulnerable populations' access to healthcare. In particular, five of these nine studies found that poverty (i.e., residing in the lower income quartile level of the study area) specifically impacted a population's access to healthcare resources. A lack of information due to language barriers and/or communication failure was also mentioned in 33% (*n* = 3) of the studies. Figure 3 displays a bar chart of identified barriers in Global North studies among the vulnerable groups.

##### 3.2.1.1 North America

All North American studies conducted in the Global North found that poverty prevented vulnerable populations from accessing healthcare resources, obtaining transportation to healthcare services, and/or led to unsafe living conditions that contributed to health inequities. Particularly, four of these studies noted that limitations to transportation due to cost was a critical barrier for populations who had to travel for care. For example, Mendez et al. (2020) found that the Thomas fire, a climate change–related disaster that occurred in California, created and reinforced barriers to transportation for vulnerable populations.

Two studies found that during natural disaster events vulnerable populations were made more susceptible to adverse health outcomes because of systemic barriers such as disaster relief policy restrictions for certain populations, disaster protocol and information communicated primarily in English, and a general lack of disaster information being shared with vulnerable populations (Albahsahli et al., 2023; Mendez et al., 2020). For example, Mendez et al. (2020) found that populations who spoke a language other than English in California were unaware of safety guidelines and evacuation protocols, which were written only in English, during natural disasters.

##### 3.2.1.2 Oceania

All Oceanic articles based in the Global North found that poverty disrupted vulnerable populations' ability to access healthcare resources and climate change preventative supplies such as air conditioners. In particular, extreme temperatures interacted with structural considerations such as workplace protocols and the grouping of impoverished neighborhoods to prevent access to healthcare. An example of this is Loughnan et al. (2010), which found that those in the most socioeconomically disadvantaged and least socioeconomically disadvantaged areas of Australia had greater rates of acute myocardial infarction.

##### 3.2.1.3 Europe

One study found that occupational hazards, language barriers, and lack of information given to vulnerable groups impacted their ability to access healthcare during exposure to increasing temperatures. For example, Salvador et al. (2023) found that cultural and/or language barriers contributed to less awareness of health‐related risks in vulnerable populations in Spain.

#### 3.2.2 Discussion: Addressing Barriers in the Global North

##### 3.2.2.1 North America

Most of the North American studies conducted in the Global North discussed poverty as a primary barrier to healthcare, specifically by preventing vulnerable populations from accessing healthcare resources. To address this obstacle in climate change mitigation strategies targeted in North America, it is critical for policymakers to consider the transportation barriers that vulnerable populations face due to cost, which can prevent them from accessing care. Recommendations to reduce transportation emissions while concurrently reducing transportation barriers include (a) enhancing land planning to make areas walkable and (b) investing in energy‐efficient forms of transportation such as public transit (U.S. Department of Transportation, 2023). Additionally, we encourage policymakers to consider our recommendations and guidelines from other leading climate justice researchers (Table 3) to more effectively tackle the distinct structural and institutional barriers faced by vulnerable populations in North America.

##### 3.2.2.2 Oceania

The majority of Oceanic studies in the Global North highlighted poverty's impact on vulnerable populations' access to healthcare resources. To address this barrier to healthcare access in climate change policies, policymakers should consider the following recommendations to mitigate urban overheating and improve access to climate preventative supplies (e.g., air conditioners): (a) utilize green and blue infrastructure in city planning and (b) allocate funds to building community resilience hubs (Grunwald et al., 2022; Kumar et al., 2024). Other recommendations for addressing the impact of climate change stressors on the health and health access of vulnerable populations in Oceania can be found in Table 3.

##### 3.2.2.3 Europe

The one study in this review that was conducted in Europe discussed the importance of accounting for cultural diversity and language differences when disseminating climate change mitigation procedures and recommendations to vulnerable populations. To effectively account for these considerations in climate change mitigation measures, global leaders in social change have suggested (a) using accessible language, (b) considering the knowledge of the target population, and (c) ensuring comprehensive dissemination (Rakhimov et al., 2022). Yore et al. (2023) provide comprehensive recommendations for good practices and entry points in designing inclusive and accessible early warning systems for climate change–related disasters. Additional resources for policymakers focused on climate change stressors in Europe can be found in our guidelines and recommendations in Table 3.

#### 3.2.3 Global South

Compared to the Global North a lack of nutritious food and clean water was a more significant barrier to health in the Global South, with 50% (*n* = 5) of studies citing this issue. For both regions, poverty was the leading cause of health access issues. Eighty percent (*n* = 8) of studies conducted in the Global South found that poverty adversely impacted vulnerable populations' ability to access healthcare, particularly in terms of being able to afford healthcare resources. Additional information about barriers faced by vulnerable populations in the Global South can be found in Figure 3.

##### 3.2.3.1 Asia

Three out of four studies found that poverty limited access to healthcare resources. Moreover, one of these studies also found that poverty limited transportation to healthcare, while another found that poverty contributed to unsafe living conditions after exposure to a climate stressor (Hu et al., 2019; Thamarapani, 2021). Region of residence was particularly significant in determining access barriers to healthcare and drivers of health inequities, since inhabiting a healthcare shortage area due to rurality (Hu et al., 2019; Lin et al., 2022), lack of nutritional resources (Kabir et al., 2016; Thamarapani, 2021), and increased locational heat exposure (Hu et al., 2019) were all found to impact the health outcomes of vulnerable populations. For example, Hu et al. (2019) found that the lack of air conditioning in rural areas in China contributed to farmers' experiencing increased heat exposure, and health concerns related to these exposures were difficult to address because they lived in rural areas. Occupational hazards are also important to note, as 50% (*n* = 2) of studies found that the nature of certain occupations and their rural locations contributed to adverse health outcomes and barriers to healthcare.

##### 3.2.3.2 Africa

All articles (*n* = 3) found that poverty adversely affected populations' ability to access healthcare resources. Out of these articles, 33% (*n* = 1) found that poverty contributed to unsafe living conditions, and 66% (*n* = 2) found that poverty limited access to transportation to healthcare. For example, MacVicar et al. (2017) found that the cost of transportation to the hospital was a crucial factor that impacted the high prevalence of home deliveries for pregnant Batwa women in Uganda. Environmental climate changes also contributed to health inequities faced by vulnerable populations, since 66% (*n* = 2) of studies found that a change in the surrounding environment limited access to clean water and nutritional foods. Sixty‐six (*n* = 2) of studies also found that climate change contributed to a loss of work or wages, which further prevented individuals from accessing healthcare and nutritional resources.

##### 3.2.3.3 South America

One study found that lack of information paired with a lack of clean water contributed to vulnerable populations experiencing adverse health outcomes. One example of this is Da Mata et al. (2023), who found that a program aimed at spreading knowledge of clean water practices and increasing access to water cisterns reduced adverse pregnancy outcomes in Brazil.

##### 3.2.3.4 North America

The one North American study conducted in the Global South found that barriers such as poverty, healthcare shortage areas, and a lack of information adversely impacted vulnerable populations' ability to access health resources. For example, Behrman and Weitzman (2016) found that poverty interacted with a lack of knowledge on where to obtain sexual health resources and a lack of places to receive healthcare to increase the likelihood that Haitian womens' family planning needs were unmet during natural disasters.

##### 3.2.3.5 Oceania

The one Oceanic study conducted in the Global South found that policy restrictions and a lack of information given during natural disasters contributed to barriers to healthcare access and adverse health outcomes (e.g., increased sexually transmitted disease). For example, Murphy and colleagues (2023) found that access to resources like food and water declined during climate change–related disasters, which increased Fijian youths' use of transactional sex to fulfill food and water insecurities.

#### 3.2.4 Discussion: Addressing Barriers in the Global South

##### 3.2.4.1 Asia

Most articles conducted in Asia for this review mentioned poverty as a main barrier to the access of healthcare resources. To address this obstacle in Asia, it is crucial that policymakers consider solutions to improve limited transportation and unsafe work conditions. The following recommendations can be utilized to address these concerns for vulnerable populations in Asia: (a) investing in clean public infrastructure; (b) funding a universal lump sum subsidy for rural households; and (c) extending unemployment benefits, training, and reemployment services (Dabla‐Norris et al., 2021). Furthermore, we recommend that policymakers review additional guidelines and resources for climate change mitigation strategies in Asia that can be found in Table 3.

##### 3.2.4.2 Africa

The majority of articles conducted in Africa highlighted the impact of poverty on vulnerable populations' access to healthcare resources. Particularly, lack of access to transportation was the greatest barrier to accessibility. To effectively address these considerations in climate change mitigation measures, policymakers can implement the following recommendations: (a) invest in climate‐resilient transportation infrastructure, (b) include disadvantaged groups in discussions about design policy and implementation, and (c) create infrastructure that supports active travel modes (e.g., cycling and walking; Cinderby et al., 2024). Additional recommendations and resources for climate change mitigation strategies in Africa can be found in Table 3.

##### 3.2.4.3 South America

The one study in this review that was conducted in South America discussed the lack of infrastructure to obtain clean water and lack of knowledge about clean water practices on the health of vulnerable populations. To account for this barrier in climate change policies, climate change leaders have suggested: (1) developing hydraulic infrastructure, (3) addressing water productivity and efficient irrigation practices, (3) improving financing to operate and maintain water sanitation services, particularly for rural populations (Wellenstein & Makino, 2022). A notable study that explores the impact of green infrastructure in South America is (Rodrigues et al. (2023), which investigates the feasibility of implementing green infrastructure in Brazil. Additional recommendations and resources for South America can be found in Table 3.

##### 3.2.4.4 North America

North American studies conducted in the Global South highlighted the need to address healthcare shortage areas at a time of climate crisis. Recommendations for addressing this need are as follows: (a) provide new insurance mechanisms to reduce the cost of care, (b) include vulnerable communities in the decision‐making process, and (c) create incentives to provide vulnerable populations with expanded health services (Guivarch et al., 2021). Furthermore, we recommend that policymakers review our considerations and guidelines from other leading climate justice researchers (Table 3) to effectively address the unique structural and institutional barriers that impact vulnerable populations in North America.

##### 3.2.4.5 Oceania

Additionally, Oceanic studies conducted in the Global South found that policy restrictions and lack of information impacted vulnerable populations' ability to access healthcare resources the most. Recommendations for enhancing community collaboration in the climate mitigation process include (a) sharing local knowledge and resources, (b) nurturing social connection with vulnerable communities, and (c) involving vulnerable populations in discussions to address their needs (Latai‐Niusulu et al., 2024). Other recommendations for addressing climate change stressors and their impact on the health and health access of vulnerable populations in Oceania can be found in Table 3.

#### 3.2.5 Differences Between the Global North and Global South

The differences in access issues and barriers between the Global North and Global South highlight the complex interplay of systemic and structural factors that contribute to healthcare inequities. In the Global North, studies predominantly identified poverty as a significant barrier, with specific systemic challenges such as transportation limitations and language barriers exacerbating the issue. For instance, in North America, the intersection of poverty and inadequate transportation can prevent vulnerable populations from reaching healthcare services, particularly during climate‐related disasters when access is further restricted. In contrast, the Global South faces more pronounced barriers related to basic needs, such as access to nutritious food and clean water, which are often cited as critical factors impacting health. Although poverty remains a leading cause of access issues in both regions, studies from the Global South emphasize the direct effects of environmental changes and resource scarcity on health outcomes. For example, a lack of clean water and inadequate nutritional resources can lead to severe health complications, particularly in rural areas where healthcare facilities are limited. This stark contrast underscores the need for tailored interventions that consider the specific geopolitical contexts and systemic challenges faced by vulnerable populations in each region.

## 4 Discussion

Reducing negative health outcomes from climate change stressors is a critical issue that must be addressed globally to align with the United Nations' call for climate mitigation effort. Particularly, the impact of climate change on vulnerable populations' health and healthcare access requires imminent attention as increased exposure to climate change stressors interact with systemic barriers to create compounded healthcare burdens for everyone, and particularly vulnerable groups. As policymakers continue to address climate change, our findings demonstrate the impact of systemic and institutional barriers on health outcomes and inequities when facing climate change stressors so that future policies can create more effective and sustainable climate change mitigation strategies. Importantly, our scoping review developed a list of considerations and recommendations gathered from climate change experts (see Table 3) for including the health needs of vulnerable populations in climate change mitigation strategies.

While our scoping review identifies crucial associations between climate change stressors, health outcomes, and access issues faced by vulnerable populations, a limitation of our review is the inability to establish causal relationships. The studies we reviewed primarily assess associations, between climate exposures and health outcomes using a variety of methods ranging from qualitative (e.g., thematic analysis, content analysis) to quantitative (e.g., *t*‐test, MANOVA, difference‐in‐difference analysis, odds ratio, meta‐regression, and etc.). These differences limit direct comparability and generalizability. Therefore, the contextual strengths and methodological constraints of each study should be considered when interpreting this review. Despite these limitations, addressing the healthcare needs of vulnerable populations such as women, children, farmworkers, rural and island communities, racial/ethnic minorities, and refugees and immigrants is crucial to reducing health inequities because these populations face oppression from governmental rules and laws that determine healthcare funding and eligibility standards for healthcare assistance (Tzenios, 2019). Furthermore, these populations are often voiceless during the development of climate change mitigation strategies and healthcare policies. For example, Grineski and collegues (2022) evaluated the implementation of citizen science efforts and how these policies were not implemented communally and inclusively, resulting in climate injustice being inadvertently reinforced and exacerbated, leaving marginalized voices further silenced and their specific needs overlooked in the broader climate discourse. Without collaborative engagement, these initiatives risk perpetuating existing inequities rather than fostering equitable solutions. Therefore, vulnerable populations are more susceptible to adverse and severe health outcomes because of unique structural and systemic barriers that prevent certain populations from accessing healthcare. By synthesizing knowledge on the access barriers and adverse climate change–related health outcomes that impact vulnerable populations, policies can more effectively address the health inequities among vulnerable populations.

## 5 Conclusion

To address rising concerns about the healthcare access of vulnerable populations affected by climate change, we urge policymakers to create more impactful climate change mitigation strategies by utilizing the recommendations our team compiled from climate change experts to more effectively address the unique needs of different vulnerable populations around the globe. It is our goal that the provided recommendations help policymakers take tangible steps to address different structural/institutional barriers that interact with specific climate change stressors. Overall, we hope to further the knowledge and understanding of how climate change stressors interact with institutional and structural barriers to produce unique healthcare access outcomes for vulnerable populations, which can then inform policy change to address the healthcare needs of these populations.

## Conflict of Interest

The authors declare no conflicts of interest relevant to this study.

## Supporting information

Supporting Information S1

## Data Availability

This paper did not analyze any new data. The information of the 21 studies included in this scoping review were listed in Table 2 and in the reference list.
