# Supplementary material for: How Systemic Barriers Can Impact Health Inequities When Facing Climate Change Stressors: A Scoping Review of Global Differences
Source: Geohealth. 2025 Jun 18;9(6):e2024GH001272. doi: 10.1029/2024GH001272 (PMC12174864; doi:10.1029/2024GH001272)
Supplement: Supplementary file 1 — Supporting Information S1 [file GH2-9-e2024GH001272-s001.docx]

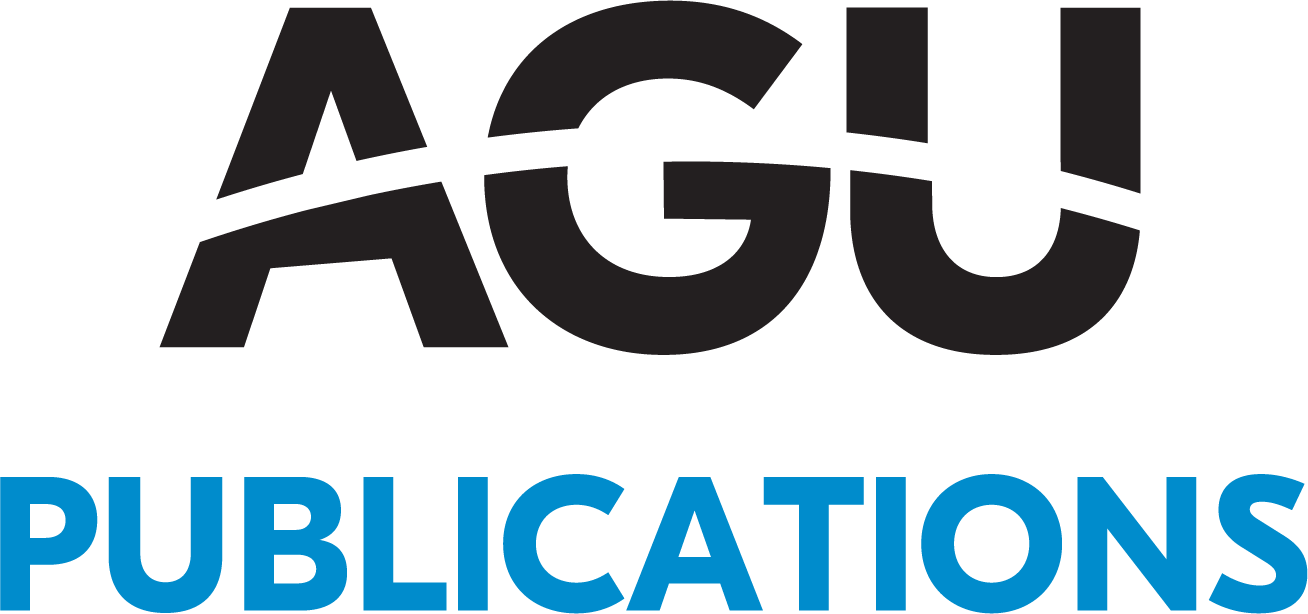


*GeoHealth*

Supporting Information for

**How Systemic Barriers Can Impact Health Inequities When Facing Climate Change Stressors: A Scoping Review of Global Differences**

Ainslee Wong,^1^ Tuyet-Mai H. Hoang,^2^ Victoria Ferrara,^2^ and Thanh H. Nguyen^3^

^1^ Department of Health and Kinesiology, University of Illinois Urbana-Champaign, Freer Hall, 906 S Goodwin Ave., Urbana, IL, 61801, USA

^2^ School of Social Work, University of Illinois Urbana-Champaign, 1010 W Nevada St., Urbana, IL 61801, USA

^3^ Department of Civil Engineering, University of Illinois at Urbana-Champaign, 205 N Mathews Ave., Urbana, IL 61801, USA

**Contents of this file**

Text S1

**Introduction**

The supporting information for this study includes the list of assessment questions used by the authors to evaluate whether the reviewed studies met the criteria for acceptable scientific rigor for inclusion in the scoping review.

Text S1.

Manuscript Information Author/Title/Year _______________________________________________

External validity

1. Was the study’s target population a close representation of the national population in relation to relevant variables?

2. Was the sampling frame a true or close representation of the target population?

3. Was some form of random selection used to select the sample OR was a census undertaken?

4. Was the likelihood of nonresponse bias minimal?

Internal validity

5. Were data collected directly from the subjects (as opposed to a proxy)?

6. Was an acceptable case definition used in the study?

7. Was the study instrument that measured the parameter of interest shown to have validity and reliability?

8. Was the same mode of data collection used for all subjects?

9. Was the length of the shortest prevalence period for the parameter of interest appropriate?

10. Were the numerator(s) and denominator(s) for the parameter of interest appropriate?

11. Summary item on the overall risk of study bias: Total _________

Notes:
